# Digital Fingerprinting of Complex Liquids Using a Reconfigurable Multi‐Sensor System with Foundation Models

**DOI:** 10.1002/advs.202407513

**Published:** 2024-10-07

**Authors:** Gianmarco Gabrieli, Matteo Manica, Joris Cadow‐Gossweiler, Patrick W. Ruch

**Affiliations:** ^1^ IBM Research Europe Säumerstrasse 4 Rüschlikon 8803 Switzerland

**Keywords:** chemical sensing, multi‐sensor systems, vision foundation models

## Abstract

Combining chemical sensor arrays with machine learning enables designing intelligent systems to perform complex sensing tasks and unveil properties that are not directly accessible through conventional analytical chemistry. However, personalized and portable sensor systems are typically unsuitable for the generation of extensive data sets, thereby limiting the ability to train large models in the chemical sensing realm. Foundation models have demonstrated unprecedented zero‐shot learning capabilities on various data structures and modalities, in particular for language and vision. Transfer learning from such models is explored by providing a framework to create effective data representations for chemical sensors and ultimately describe a novel, generalizable approach for AI‐assisted chemical sensing. The translation of signals produced by remarkably simple and portable multi‐sensor systems into visual fingerprints of liquid samples under test is demonstrated, and it is illustrated that how a pipeline incorporating pretrained vision models yields >95% average classification accuracy in four unrelated chemical sensing tasks with limited domain‐specific training measurements. This approach matches or outperforms expert‐curated sensor signal features, thereby providing a generalization of data processing for ultimate ease‐of‐use and broad applicability to enable interpretation of multi‐signal outputs for generic sensing applications.

## Graphical abstract

Translating signals produced by portable multi‐sensor systems into image representations enables the generation of visual fingerprints of liquid samples under test. A transfer learning pipeline incorporating vision foundation models pretrained on natural images yields >95% average classification accuracy when applied to visual chemical sensor fingerprints in four unrelated sensing tasks with limited domain‐specific training measurements.

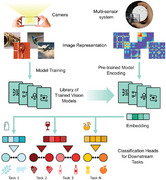

## 1 Introduction

The combination of machine learning and chemical sensing to enhance human perception and robotics has the potential to benefit society in key domains, including water and food safety,^[^ ^1^, ^2^, ^3^ ^]^ sustainable agriculture,^[^ ^4^ ^]^ healthcare,^[^ ^5^, ^6^, ^7^ ^]^ enhanced human–machine interactions^[^ ^8^, ^9^, ^10^ ^]^ and materials discovery.^[^ ^11^ ^]^ Concomitantly, the use of ubiquitous, low‐cost, and open sensing hardware has been shown to facilitate access to scientific research systems at scale.^[^ ^12^ ^]^ Advances in data processing along with improved and more accessible sensing technologies have contributed to the democratization of chemical analysis, whereby portable sensors can be used by any individual or artificial system to perform increasingly complex sensing tasks.

A formidable challenge in unlocking the utility of chemical sensors is the ease of configuration for a particular application. Tailoring sensors according to the “lock‐key” design makes such systems vulnerable to compositional variations of the chemical background, preventing broad applicability and often introducing the burden of task‐specific sample preparation in order to obtain useful results.^[^ ^13^ ^]^ Indeed, the human senses of olfaction and taste comprise open systems of noteworthy breadth and discriminatory power based on collective assessments of multiple sensory receptors.^[^ ^14^, ^15^, ^16^, ^17^ ^]^ Efforts to incorporate the advantages of physiological chemical senses into artificial systems have spawned many varieties of electronic tongues and electronic noses that evaluate multiple complementary signals using machine learning to benefit applications in food, health, agriculture, and robotics.^[^ ^10^, ^18^, ^19^, ^20^, ^21^, ^22^, ^23^ ^]^ For example, arrays of graphene field‐effect transistors have been coupled with artificial neural networks and tree‐based algorithms to account for cross‐sensitivity during detection of toxins and cations in water.^[^ ^24^, ^25^ ^]^ Metal and ZnO‐based liquid and gas sensors have been combined to design a portable personalized gustatory and olfactory system supported by machine learning models for coffee bean analysis.^[^ ^26^ ^]^ Moon et al. reported the on‐chip integration of a chemoresistive sensor array for NO_*x*_ monitoring and sensitivity estimation by leveraging classification models.^[^ ^27^ ^]^

Today, data collected by electronic tongues and noses is commonly evaluated with classical pattern recognition and machine learning algorithms,^[^ ^20^ ^]^ and in some cases leveraging deep learning architectures based on low‐dimensional Convolutional Neural Networks (CNNs).^[^ ^28^, ^29^, ^30^, ^31^ ^]^ Two interrelated key challenges in generalizing such systems are the effective featurization of the data as well as the scarcity of training data, which are exacerbated in view of larger, more proficient deep learning models with higher parameter count and the heterogeneity of measurement data produced by different types of sensor arrays. Classical machine learning algorithms, such as Support Vector Machines (SVMs) or Random Forest (RF), rely on the definition of handcrafted features by experts, while deep learning algorithms such as CNNs learn data representations from the input data.^[^ ^32^ ^]^ Effective representation learning requires sufficiently abundant and representative training data, which is often difficult to obtain or generate in the chemical sensing domain. The pursuit of representation learning in chemical sensing has led to the exploration of transfer learning, which leverages learning on more abundant data from an auxiliary domain and then transfers this information to a target domain.^[^ ^33^ ^]^ Yang et al. pretrained a CNN model on a large speech recognition dataset relying on similarities between audio signal waveforms and pulsed voltammetry signals from an electronic tongue as sparse target domain data.^[^ ^34^ ^]^ Cadow et al. employed deep learning models trained on natural images to process liquid chromatography/tandem mass spectrometry (LC‐MS‐MS) data for the classification of clinical tissue samples.^[^ ^35^ ^]^ Zhang et al. pretrained deep learning models on Raman spectra from databases of organics and minerals, and demonstrated improved performance for classification of unseen organic compounds.^[^ ^36^ ^]^ While this previous work has described promising approaches for addressing data scarcity challenges in chemical sensing, the sheer variety of sensing devices and measurement methods still poses a major problem for the identification of appropriate learning domains and methods to enable the widespread application of deep learning to open and portable chemical sensing systems.

Foundation models represent a recently emerged paradigm for developing machine learning systems, in which deep neural networks based on the transformer architecture^[^ ^37^ ^]^ are pretrained on broad data at scale, usually on unlabeled data under self‐supervision, and adapted to a wide range of downstream tasks.^[^ ^38^ ^]^ Large vision models such as ViT^[^ ^39^, ^40^, ^41^ ^]^ have demonstrated increasing performance with scale while requiring substantially fewer computational resources to train and advance state‐of‐the‐art results on image benchmarks. Leveraging foundation models for chemical sensing is a novel concept that requires appropriate representation of the input data. Typically, vectorization approaches are applied to prepare numeric data for deep learning in analytical chemistry.^[^ ^42^ ^]^ For example, Raman spectra have been used to train CNNs for classification by providing the fully sampled intensity at regularly spaced wavenumbers as 1D inputs.^[^ ^43^, ^44^ ^]^ 1D Fourier transforms can be used to convert time series electrical resistance data from gas sensors to the frequency domain for training of fully connected autoencoders.^[^ ^45^ ^]^ However, the lack of domain‐specific datasets has so far precluded the investigation of large, pretrained models for applications in chemical sensing.

In the present contribution, we explore an approach to close the gap between large pretrained foundation models and low‐cost, open, portable multi‐sensor systems for chemical sensing that is agnostic to the format and structure of the sensor output data. The proposed method encodes multi‐sensor outputs into image representations, thereby producing visually discernible digital fingerprints of samples under test. We demonstrate that such fingerprints are amenable to effective featurization through pretrained computer vision models, thereby enabling diverse chemical sensing tasks to be addressed while circumventing feature handcrafting for different sensing modalities.

We use a portable multi‐sensor potentiometric system^[^ ^46^ ^]^ to study the proposed concept during accelerated chemical analysis of liquids. First, we demonstrate the discrimination of liquid samples by combining image encoding of multi‐sensor data with learnt representations from four pretrained vision models representing CNN‐based (ResNet^[^ ^47^ ^]^ and MobileNetV2^[^ ^48^ ^]^) and transformer‐based (ViT^[^ ^39^ ^]^ and BeiT^[^ ^49^ ^]^) architectures. These models represent the state‐of‐the‐art of the respective architecture type, and are capable of generating effective representations of natural images due to their pretraining on very large datasets. Then, we explore the use of these embeddings to train classification heads for four different exemplary analysis tasks: discriminating types of red wine, identifying the origin of plant‐based protein and milk, and recognizing the type and abundance of sugar in solution. Gramian Angular Field (GAF) image representations are evaluated with two different approaches to conserve information during encoding of the sensor data. We compare the classification accuracy with that obtained through previously reported handcrafted feature representations. Finally, we present potential benefits of the proposed featurization approach for data augmentation and few‐shot learning, paving the way for effective application of foundation models to multi‐sensor chemical analysis in scenarios suffering from a scarcity of training data.

## 2 Results

### 2.1 Transfer Learning for Chemical Sensing

**Figure 1:**
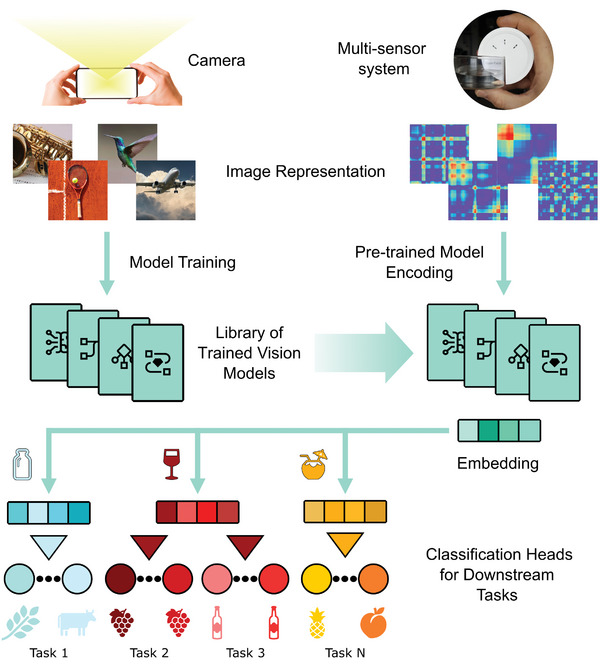
Overview of transfer learning leveraging vision models for chemical sensing. Model architectures trained on large datasets containing millions of images are transferred to featurize image sets from the chemical sensing domain. The responses of multi‐sensor systems are encoded as images to enable effective feature extraction by means of pretrained vision models. The resulting embeddings represent liquid fingerprints that are used to train classification heads and perform downstream tasks.

**Figure 2:**
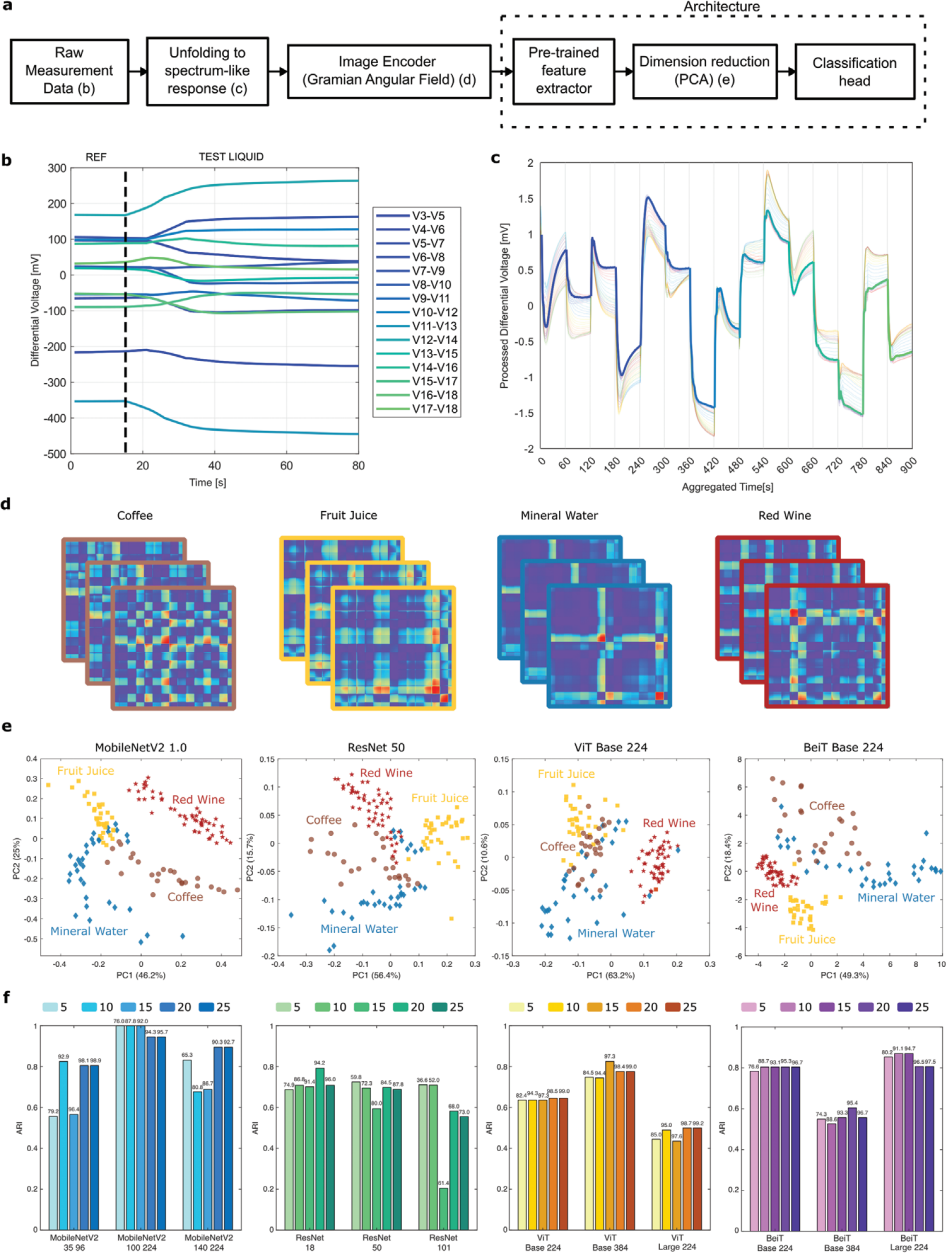
Overview of encoding and learning pipeline. a) Schematic diagram of data pipeline from raw measurement data acquisition to signal encoding into image representation and model architecture. Feature extraction with pretrained vision models is followed by Principal Component Analysis (PCA) and then training of the classification model heads. b) Example of raw data acquisition during transition of a model sensor array from reference (mineral water) to test liquid (coffee sample) giving rise to perturbation of 15 differential voltages. c) Example of raw measurement data unfolding to form continuous spectrum‐like responses for repeated measurements of eight coffee samples. d) Example of image encodings obtained for four different sample classes after Gramian Angular Field (GAF) encoding. e) Principal component plots of features extracted by pretrained vision models from measurements of mineral water, red wine, fruit juice and coffee samples. f) Adjusted Rand Index (ARI) of agglomerative clustering for twelve pretrained vision models with increasing number of principal components (5, 10, 15, 20, and 25) as input features. The percentage of explained variance is reported at the top of each bar.

The transfer learning pipeline comprises vision models that are pretrained on natural images and can be used subsequently with frozen weights as feature extractors for chemical sensor array data (**Figure** **1**). Thus, the raw measurements of any multi‐sensor system are preprocessed and encoded into image representations prior to being provided to the pretrained vision models. The model pooled outputs are numeric vectors representing fingerprints associated with each liquid sample being tested. The high dimensionality of the fingerprint vectors generated by the vision models is then reduced from >1000 to ⩽25 through Principal Component Analysis (PCA), and a set of principal component scores is input to different classification heads for chemical sensing tasks. The training data is obtained by exposing the sensor array to a set of training liquids, the examples that are associated to a task‐specific chemical space. The system is reconfigured for different sensing tasks by training the classification heads with measurement data from samples in a specific domain, without any changes to the vision models used for image encoding. The end‐to‐end pipeline to enable transfer learning for chemical sensing is presented in **Figure** **2**.

### 2.2 Multi‐Sensor Model System

**Figure 3:**
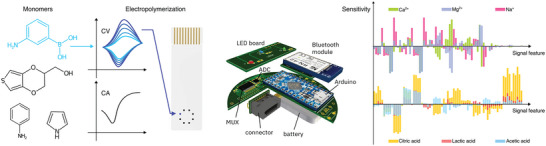
Multi‐sensor model system. The multi‐sensor system used in the study comprises an array of 16 conductive polymers whose base monomers are pyrrole, aniline, 3‐aminophenylboronic acid (APBA) or 3,4‐ethylenedioxythiophene (EDOT), and are electrodeposited by chronoamperometry (CA) or cyclic voltammetry (CV) on electroless nickel immersion gold (ENIG) pads of a double‐sided printed circuit board (PCB). The PCB sensor array is connected to a battery‐powered microprocessor assembly comprising two 16‐channel multiplexers (MUX) and an analog‐to‐digital converter (ADC) to enable potentiometric data recording and transmission via wired or wireless serial communication. In previous studies, the array has been demonstrated to be cross‐sensitive, as the same signal characteristics respond to multiple ionic and organic species with varying sensitivity.

The model multi‐sensor system used in the present study comprises an array of 16 miniaturized polymeric sensors as described in ref. [46]. The sensor array is fabricated by electrodeposition (cyclic voltammetry or chronoamperometry) of polypyrrole (PPy), poly(3,4‐ethylenedioxythiophene) (PEDOT), polyaniline (PANI) and poly(3‐aminophenylboronic acid) (PAPBA) conductive polymers enriched with doping agents (**Figure** **3**). The different polymers and dopants were chosen to introduce complementary affinities toward charged and neutral small‐molecule analytes. The array is integrated with a robotic platform for automated testing of liquid samples as described in ref. [50]. Each liquid sample is tested by immersion of the sensor array in a reference liquid for 100 s followed by immersion in a test liquid for 60 s. At the end of each test, the sensor is dipped in a rinsing liquid for 10 s in order to rinse the sensor substrate and reduce sample carry‐over. Potentiometric time‐series data is acquired for each test sequence by recording 15 differential voltages at a rate of 1 Hz and data transmission via wired (USB) or wireless (Bluetooth) serial connection to a PC.^[^ ^50^ ^]^ Previous studies^[^ ^51^, ^52^ ^]^ confirmed the cross‐sensitivity of the sensor array in multi‐component media, whereby the same polymeric sensor exhibits enhanced sensitivity to multiple analytes. The multi‐sensor system therefore represents a suitable model platform to explore transfer learning and foundation models for chemical sensing, whereby the findings can be applied to any multi‐sensor system that produces multiple output signals in response to interaction with a chemical sample.

### 2.3 Visualization of Chemical Fingerprints

Appropriate representation of multi‐sensor data is crucial to enable the use of pretrained vision models. Such data consists of multiple signals, whose perturbation depends on the sensing transduction mechanism and may result in a time‐dependent response or in a signal evolution induced by an external parameter sweep. We propose a generic approach to generate image representations from multi‐sensor data (cf. Experimental Section; Figure S1, Supporting Information) by concatenating raw signals and applying the Gramian Angular Field (GAF) transformation. GAF has been proven to be an effective strategy to obtain image representations of raw vector data, as it features a smaller uncertainty in reconstruction of the original data through inverse operations than other approaches.^[^ ^53^, ^54^ ^]^ This helps preserve correlations between data recorded within the same sensor and across multiple sensors. An example of raw data recorded with the model multi‐sensor system is shown in Figure 2b, in which the sensor array is exposed successively to a reference liquid (mineral water) and a test liquid (coffee), leading to an electrochemical perturbation of the sensor responses. The sensor signals are then concatenated to form a spectrum‐like response and the same procedure is repeated for all measurements in the data set (Figure 2c). The concatenated time‐series data is converted through the GAF transformation to obtain a collection of images as visual fingerprints of the tested samples. Example images for four different liquid categories exhibit distinct visual patterns depending on the type of liquid being tested (coffee, fruit juice, mineral water, and red wine, cf. Figure 2d), motivating their use as input images for pretrained vision models.

### 2.4 Vision Models Produce Discriminatory Representations of Liquid Samples

The visual fingerprints of liquid samples are featurized using pretrained vision models to obtain vector representations of the liquids under test. Each of the four investigated vision model architectures produces distinct representations of the multi‐sensor data as demonstrated by the principal component plots for repeated measurements of multiple samples belonging to four different liquid categories (PC1‐PC2 projections, Figure 2e). The principal component plots illustrate at the same time the effective featurization of sensor data and the different ways in which the discrimination of liquids occurs depending on the model architecture that was used. Unsupervised cluster modeling was performed to quantify the discriminating capabilities of the prelearnt representations for all model architectures investigated in our work (Figure 2f). The Adjusted Rand Index (ARI) of agglomerative clustering generally takes values greater than 0.4, with only one exception for *ResNet 101*. *MobileNetV2 100 224* scores perfect clustering for all the tests performed with the multi‐sensor system using 5, 10, or 15 principal components. Effective clustering results can already be obtained with smaller model versions and lesser number of principal components.

### 2.5 Liquid Fingerprints Enable Product Identification

**Figure 4:**
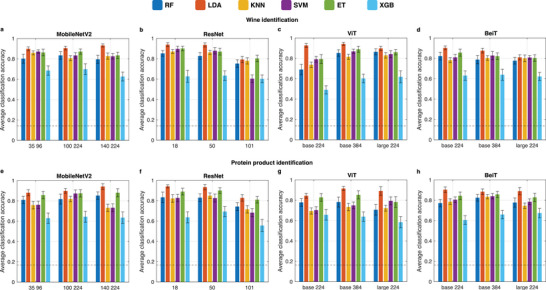
Results on product identification tasks. Fivefold cross‐validation classification accuracy for wine (a–d) and protein product (e–h) identification using 12 distinct pretrained vision models as feature extractors. In each case, the features are reduced to 20 principal components and used to train six different classification model heads: Random Forest (RF), Linear Discriminant Analysis (LDA), K‐Nearest Neighbors (KNN), Support Vector Machine (SVM), Extra Trees (ET) and XGBoost (XGB). The dashed lines represent accuracy achieved with a random predictor used as model baseline and scoring 14.3% and 16.7% for wine and protein datasets, respectively.

Liquid discrimination leveraging the pretrained vision models with multi‐sensor systems is demonstrated for two specific use cases: identification of a wine sample among a set of seven bottled Italian red wines, and classification of the source of a protein product among a set of six plant‐derived protein products from different plants (pea or soy) and vendors. The average classification accuracy exceeds 80% for each of the twelve vision models for at least one classification head, based on five‐fold cross‐validation using the top 20 principal components of the prelearnt representations produced by the vision models (**Figure** **4**). For both experiments, the best accuracy is achieved with LDA as classification head while the XGB model performed consistently worst, regardless which of the twelve vision models was used as feature extractor. For the wine experiments (Figure 4a–d), the average classification accuracy varies between 49.1% and 94.3% across all model configurations (details in **Table** **1**). For the *MobileNetV2* feature extractor, image and model size do not substantially affect the classification accuracy. Instead, *ResNet 101* with the largest number of layers reaches the lowest accuracy scores among the *ResNet* models, with only one model head (ET) reaching 80% average accuracy. Regarding the transformer architectures, *ViT*, combined with the LDA classification head, achieves the best average accuracy (94.3%) across all model configurations. While for *ViT base 224* only the LDA model enables high prediction accuracy, the scores obtained with *ViT base 384* and *ViT large 224* exceed 80% average accuracy for all classification heads except XGB. For the *BeiT* architectures, *BeiT base 224* achieves better average accuracy than its *ViT* counterpart using RF, KNN, SVM, ET and XGB model heads. Instead, *ViT* generally demonstrates superior accuracy with increasing image resolution and architecture size for most model heads.

**Table 1:** Benchmarking of classification accuracy across featurization methods and models. Comparison of average classification accuracy for wine fingerprinting using handcrafted expert features and fingerprints obtained from pretrained vision models as feature extractors (fivefold validation and 20 principal components). Results are presented for two image encoding approaches (GAF and GAF* as explained in the Experimental Section). MN = MobileNetV2, RN = ResNet, VB = ViT base, VL = ViT large, BB = BeiT base, BL = BeiT large. Results within the standard deviation uncertainty of the top accuracy are highlighted in bold for each classification head.

Features		RF	LDA	KNN	SVM	ET	XGB
Expert‐curated		**89.0** ± **3.2**	**99.8** ± **0.3**	59.8 ± 3.9	27.0 ± 4.7	**93.5** ± **2.1**	**75.3** ± **4.1**
GAF	MN 35 96	80.3 ± 4.4	89.9 ± 1.9	86.0 ± 2.0	**87.2** ± **1.7**	86.3 ± 3.2	68.6 ± 4.5
	MN 100 224	83.5 ± 3.7	90.6 ± 1.8	80.7 ± 2.4	83.4 ± 2.8	87.2 ± 2.5	69.9 ± 5.3
	MN 140 224	79.6 ± 3.9	93.2 ± 1.6	82.7 ± 3.0	82.6 ± 2.8	83.5 ± 3.2	62.7 ± 4.4
	RN18	85.3 ± 2.8	93.9 ± 1.9	87.1 ± 2.0	**89.7** ± **2.7**	**90.1** ± **2.3**	62.7 ± 5.8
	RN50	82.7 ± 3.3	93.6 ± 2.0	86.3 ± 2.2	**87.9** ± **3.1**	87.1 ± 3.4	63.4 ± 4.7
	RN101	75.3 ± 3.7	79.3 ± 3.0	77.9 ± 3.0	60.4 ± 3.7	80.3 ± 3.2	60.3 ± 3.8
	VB224	69.0 ± 5.1	92.7 ± 1.9	73.7 ± 2.7	79.1 ± 2.8	79.3 ± 4.3	49.1 ± 4.0
	VB384	85.3 ± 3.1	94.3 ± 1.6	81.6 ± 2.6	**86.9** ± **2.2**	**88.9** ± **2.9**	60.3 ± 4.3
	VL224	**86.5** ± **3.0**	89.8 ± 2.2	83.0 ± 2.7	**84.2** ± **3.4**	86.1 ± 2.7	61.9 ± 5.9
	BB224	82.3 ± 3.8	90.3 ± 1.9	78.3 ± 2.4	81.0 ± 2.9	85.6 ± 3.4	63.1 ± 4.6
	BB384	78.8 ± 4.1	87.6 ± 2.3	80.4 ± 2.3	82.8 ± 4.1	82.2 ± 3.3	63.9 ± 5.4
	BL224	77.7 ± 3.3	81.0 ± 2.6	80.0 ± 2.3	80.9 ± 1.9	80.3 ± 3.2	62.3 ± 4.0
GAF*	MN 35 96	**88.1** ± **3.9**	97.7 ± 1.2	**88.5** ± **2.6**	**87.3** ± **3.4**	**92.8** ± **2.4**	68.6 ± 4.2
	MN 100 224	**86.5** ± **3.9**	97.9 ± 1.3	**87.0** ± **3.4**	80.9 ± 3.6	**91.3** ± **2.3**	**72.1** ± **4.1**
	MN 100 224	84.9 ± 2.9	91.8 ± 2.3	83.8 ± 2.3	81.1 ± 2.5	86.8 ± 2.1	65.9 ± 3.8
	RN18	**87.0** ± **3.4**	96.0 ± 1.8	87.0 ± 2.7	77.8 ± 3.5	**91.5** ± **2.2**	69.5 ± 4.1
	RN50	84.0 ± 3.7	92.2 ± 2.4	84.3 ± 2.9	71.1 ± 3.0	**89.3** ± **2.3**	68.2 ± 4.3
	RN101	72.6 ± 3.4	79.5 ± 3.6	73.3 ± 3.6	40.0 ± 3.7	76.3 ± 3.4	56.3 ± 3.8
	VB224	80.5 ± 4.0	95.3 ± 1.6	81.5 ± 2.5	**90.9** ± **2.2**	88.3 ± 2.5	68.2 ± 4.4
	**VB384**	**91.5** ± **2.9**	**99.5** ± **0.7**	**92.0** ± **2.3**	**92.1** ± **2.8**	**93.5** ± **2.2**	63.8 ± 5.4
	VL224	81.0 ± 5.0	96.7 ± 1.4	73.8 ± 3.2	70.9 ± 2.8	**88.8** ± **2.8**	66.3 ± 5.0
	BB224	**86.7** ± **3.9**	93.2 ± 2.6	82.3 ± 3.4	76.6 ± 3.8	**91.6** ± **3.2**	70.5 ± 4.2
	BB384	**86.4** ± **2.9**	91.8 ± 2.2	79.6 ± 3.0	**86.0** ± **3.3**	87.7 ± 3.1	**71.1** ± **4.6**
	BL224	85.7 ± 2.2	91.0 ± 2.3	81.6 ± 2.8	83.0 ± 3.4	87.3 ± 2.4	**78.3** ± **3.6**

For protein source identification, the best average classification accuracy with respect to the model heads is obtained for LDA with 89.5%, while XGB yields the lowest accuracy with 63.4%. Similar to the wine identification use case, featurization with *ResNet 101* is the least effective (Figure 4e–h). *MobileNetV2 140 224* and *ResNet 18* combined with LDA yield the best average accuracy with 94.2%. LDA classification heads trained with features extracted by vision transformer models exhibit average classification accuracies ranging between 84.4% and 91.6%, with *ViT base 224* scoring the lowest accuracy and *ViT base 384* scoring the highest. Despite pretraining of the vision encoders on natural images (ImageNet), we find that they can produce effective representations of synthesized multi‐sensor image data. However, deeper CNN‐model architectures, such as *ResNet 101*, may produce more spurious embeddings as they tend to capture increasing complexity and granularities that may not be reflected in samples obtained in domains beyond the training space. On the contrary, this effect is less evidenced for transformer architectures, for which *large* models produce results comparable to their *base* versions. Moreover, increasing image resolution to 384 × 384 yields comparable or improved classification results for vision transformers. The enhanced performances of LDA compared to the other classification heads suggests linear separation of products in the principal component space as well as higher robustness of the model to noisy features and overfitting, which seem to affect significantly the XGB performances.

### 2.6 Liquid Fingerprints Allow Sample Categorization

**Figure 5:**
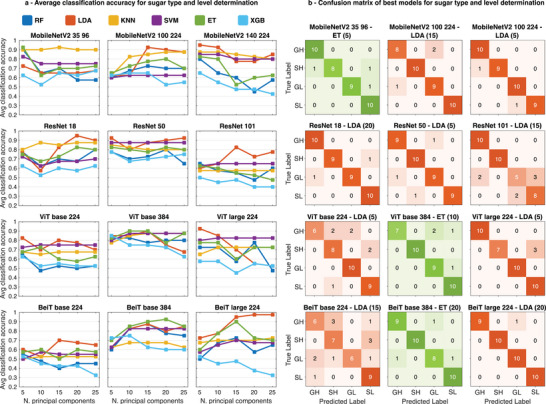
Results on sugar type and concentration level determination. a) Average classification accuracy using twelve pretrained vision models as feature extractors and six classification model heads (RF, LDA, KNN, SVM, ET, and XGB) after PCA dimension reduction (5, 10, 15, 20, and 25 principal components). b) Confusion matrices for best classification accuracy obtained with the lowest number of principal components for each of the feature extractors. GH = Glucose High, SH = Sucrose High, GL = Glucose Low, and SL = Sucrose Low.

The ability of the proposed pipeline to assign samples to predefined categories is tested through two additional tasks in which data representations obtained by vision models are used to train classification heads and assign a liquid sample to a specific group. In the first task, the objective is to identify the type and concentration level of sugars dissolved in mineral water. Multi‐sensor data is collected from glucose and sucrose powder samples dissolved in mineral water at three different concentrations (2.5, 5 and 10 wt%) and processed by models that are trained and tested as reported in the Experimental Section. The type and concentration level of an *unknown* sample can be determined with best accuracy ranging between 70% (*BeiT base 224*) and 97.5% (*BeiT large 224*) leveraging the twelve feature extractors (**Figure** **5**). Similar to the product identification tasks reported above, LDA tends to produce the highest accuracy score for nine of the vision models. For *MobileNetV2 35 96*, *ViT base 384*, and *BeiT base 384*, the ET classifier yields superior performance using 5, 10, and 20 principal components. Analogous to the product identification tasks, both the *ResNet 101* vision model for feature extraction and the XGB model head produce poorer average classification accuracy, confirming our analysis and observations for sample categorization tasks as well. Using features extracted with *ResNet 101*, the best model (LDA with 15 PCs) is not able to resolve the type of sugar in the low concentration category. Moreover, *BeiT base 224* generates the least effective representations for this task as the highest accuracy is only 70.0%, achieved with the LDA model classification head. Instead, the best accuracy score for the sugar type and concentration category across all configurations is obtained with the features extracted by *BeiT large 224* in combination with the LDA model classification head after reduction to 20 principal components. In this case, only one misclassification occurs out of the 40 test samples, yielding an accuracy of 97.5%. The number of principal components of the features used to train the model head affects the performance differently depending on the choice of feature extractor and model head. The configuration that yields the best accuracy with only five principal components is *MobileNetV2 140 224* combined with the LDA model head. The confusion matrices show that the two architectures that reached the lowest classification accuracy scores (*ViT* and *BeiT base 224*) perform poorly in discriminating the sugar type in high concentrated samples, while the low sucrose content is generally well captured by all model configurations.

**Figure 6:**
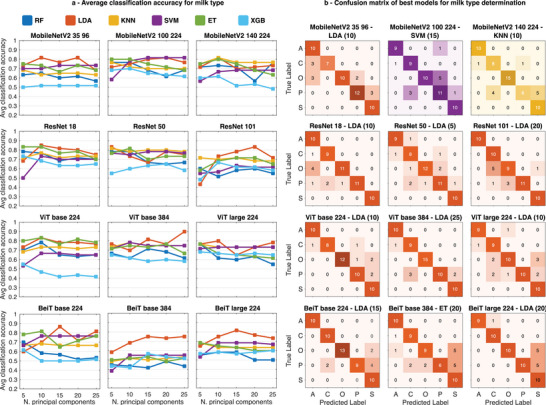
a) Average classification accuracy for determining milk type using twelve pretrained vision models as feature extractors and six classification model heads (RF, LDA, KNN, SVM, ET, and XGB) after PCA dimension reduction (5, 10, 15, 20, and 25 principal components). b) Confusion matrices for best classification accuracy obtained with the lowest number of principal components for each feature extractor (A = Almond, C = Cow, O = Oat, P = Pea, and S = Soy).

The objective of the second categorization task is to assign the correct source of commercial milk samples (cf. Experimental Section). This task proves to be more challenging with best accuracy ranging between 75.0% (*BeiT base 384*) and 88.9% (*ViT base 384*) leveraging the 12 feature extractors for the recognition of the correct sample provenance among the five classes (almond, cow, oat, pea, and soy), as shown in **Figure** **6**. The LDA classification head achieves the top accuracy for ten out of the 12 vision models. Generally, varying the number of extracted principal components does not systematically improve or worsen classification performance. The configuration yielding the highest accuracy of 88.9% (6 mispredictions over 54 measurements) is the *ViT base 384* feature extractor, combined with dimension reduction to 25 principal components and LDA model head. Compared to other tasks, the differences among feature extractors is less pronounced, with more challenges encountered by the models to resolve samples and assign them to the correct group. Poorer performances were expected due to the type and number of samples included in the data set, with only five samples per class of milk compared to ten replicates of each sample class in the other data sets. Indeed, expert‐curated features produced equal or worse results with all classification heads as reported in the next sections. Note that we find enhanced accuracy scores for milk source categorization of up to 95.0% (Table S2, Supporting Information) through the alternative image encoding scheme GAF* described in the Experimental Section and discussed below.

### 2.7 Comparing Vision Embeddings and Expert‐Curated Features

To evaluate the effectiveness of liquid fingerprints generated through vision models, we compare them to state‐of‐the‐art processing methods. For the multi‐sensor system used in the present work, we refer to a set of expert handcrafted signal characteristics designed to capture salient information from the response of an array of potentiometric sensors^[^ ^46^, ^50^ ^]^ (cf. Experimental Section). We compare the classification performance obtained in the present work through vision model embeddings with that achieved through expert features in Table 1 and Tables S1 and S2 (Supporting Information). Leveraging the GAF image encoding strategy and the classification pipeline presented in the previous sections, we obtain performances comparable to expert‐curated feature processing for the wine identification task. Our vision embedding method yields consistently better accuracy than handcrafted features when using KNN and SVM classification heads with top accuracy of 87.1% and 89.7% compared to 59.8% and 27.0%, while expert features outperform our method with LDA and XGB model heads with 99.8% and 75.3% compared to 94.3% and 69.9%. For the tree‐based algorithms RF and ET, the top accuracy obtained with pretrained vision model feature extraction equals the accuracy scores of the expert curation within the standard deviation of the average accuracy from repeated cross‐validation splits. A variation of the image encoding strategy, GAF*, was tested in order to extend the temporal correlation of the concatenated signal with information from the potentiometric signal magnitude by scaling the image representations obtained by GAF with the intensity of the raw sensor data (cf. Experimental Section). Average classification accuracy using GAF* tends to be equal or higher than for GAF for all configurations of pretrained vision model extractors and classification model heads. Thus, when compared to average scores obtained with handcrafted features, GAF* encoding proves equal or superior performances for all classification heads. In particular, *ViT base 384* yielded the best performances overall for wine classification. Regarding the CNN architectures, *MobileNetV2 35 96* and *MobileNetV2 100 224* achieved the best performances on GAF* image representations with four out of the six classification heads matching or exceeding classifiers trained on handcrafted features.

Similar observations can be made for the other classification tasks (Tables S1 and S2, Supporting Information). While protein products are generally recognized with lower accuracy, coupling GAF* encoding with KNN and SVM, classification results are better than expert‐curated features modeling and the *ViT base 384* combined with LDA achieved the top performance of 95.5%, equaling the best performance obtained with handcrafted features within the standard deviation of the performance results. Our findings suggest that multi‐sensor signals in which both temporal and amplitudal characteristics convey information about samples under test benefit from a modified encoding scheme such as GAF* over approaches that mainly emphasize temporal self‐correlation. Moreover, our approach is generalizable and can be applied to any physical system for analysis of multi‐component media.

### 2.8 Model Training with Few Examples

**Figure 7:**
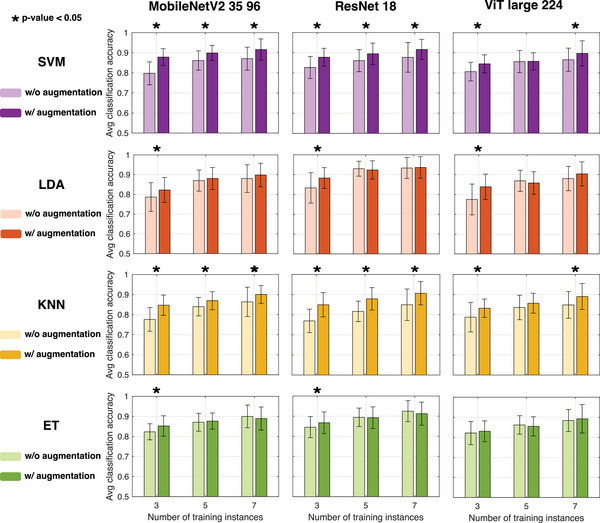
Data augmentation for multi‐sensor training. Average classification accuracy for wine identification for four classification heads trained on 10 principal components of features extracted by selected pretrained vision models (*MobileNetV2 35 96*, *ResNet 18*, and *ViT large 224*). The number of training instances correspond to the number of physical data collection events, while the columns show the difference in accuracy when the collected data is augmented by synthetic image generation by class mixing. Stars indicate instances of column pairs for which the *p*‐value obtained from a two‐sided *t*‐test is <0.05.

Our results confirm the validity and effectiveness of our approach for fingerprinting of samples to provide product identification and categorization based on pre‐defined user labels. Thus, we envision the possibility to combine sensing systems with our pipeline to ultimately enable multi‐sensor reconfiguration and personalization based on selected measurement data collected by even a single user. However, training of machine learning models in scarce data environments is a crucial challenge for chemical sensors, which is exacerbated for portable and multi‐sensor systems. We study how our pipeline can alleviate the burden of data collection by proposing a data augmentation strategy based on creating synthetic examples through image mixing from class pairs (cf. Experimental Section; Figure S4, Supporting Information). When applied to a few‐shot study on the wine identification task, we found that data augmentation generally improves the classification accuracy when using a low number of training instances (**Figure** **7**). Varying the class‐related bias in generating artificial training data via a *mixing ratio* parameter does not significantly affect the performance for three‐shot model training (Figure S5, Supporting Information). The average classification accuracy using only three training instances per class increases consistently for all configurations when augmenting the model training with synthetic samples, although in one particular configuration (*ViT large 224* with ET model head) the improvement is not statistically relevant. In the case of SVM and KNN model heads, the accuracy improvement is always statistically significant except in one case (five‐shot training in *ViT large 224*). Instead, for LDA and ET model heads, the accuracy improvement is only significant in three‐shot scenarios. Overall, SVM and KNN experienced the highest improvements for three‐shot experiments with image augmentation reaching accuracy scores in the ranges 84.5–87.8% and 83.2–84.9%, comparable to those obtained when training the models with seven‐shots without image augmentation. We conclude that image representations of chemical multi‐sensor data are amenable to data augmentation by synthetic image generation, thus offering a means to achieve higher classification accuracy in scenarios with extreme data scarcity.

## 3 Discussion

The work presented herein demonstrates that pretrained vision models can pave the way for AI‐assisted chemical sensing to leverage learnt data representations without the need for handcrafted features. We prove that, by leveraging vision foundation models, it is possible to obtain digital fingerprints of multi‐component media, which can then be used to address a variety of sensing tasks. We propose an approach to encode multi‐sensor data into image representations for featurization by pretrained models without the need for handcrafted features. Thus, the method is agnostic to the sensor transduction mechanism and data modality, and is expected to be broadly applicable to different sensing technologies. Our experiments highlight the efficacy of transfer learning from vision foundation models trained on more than one million natural images (ImageNet) to visual representations of chemical sensor measurements.

We find that, for a model system comprising an integrated potentiometric multi‐sensor system, processing of the sensor response by means of the proposed pipeline provides classification models that can be readily configured in the context of a portable sensing system to enable discrimination of various liquid types. Our results demonstrate that the embeddings extracted with vision models enable product identification and sample categorization with an average accuracy comparable to handcrafted expert‐curated features, which were previously optimized to process signals obtained with the same multi‐sensor system. In particular, transformer‐based architectures provided embeddings that yield the best performance in three out of four sensing tasks. We tentatively attribute this to the attention of transformer‐based models to global dependencies and correlations in images. Since the visual fingerprints generated from chemical sensing data tend to lack the contours expected by CNN‐based architectures that focus on isolated image patches, we hypothesize that transformer‐based models may generally be better suited for creating representations of such data. We find that the measurement embeddings of different products or groups tend to be linearly separable based on their variance, as highlighted by the high performance obtained with the LDA classification heads trained with principal component scores.

Encoding sensor data into visual representations allows practitioners to unleash image augmentation strategies in the chemical sensing domain, thus counteracting the data scarcity that prevents the extensive use of deep learning methods. We find the mixed‐image data augmentation strategy to be effective in producing useful synthetic data for model training, which is tentatively attributed to the chemical similarity within a group of samples represented by similar image patterns with varying pixel intensity. Combining the proposed method with synthetic image samples generation can increase the size of available datasets to yield higher accuracy in scenarios with few training measurements being provided. Ultimately, leveraging these methods can facilitate the training and configuration of portable multi‐sensor systems for personalized applications, making AI‐assisted chemical sensing more broadly accessible. By demonstrating the applicability of the concept within exemplary sets of complex liquids such wine, milk, protein powders, and mineral water, it is viable to extend the approach to other samples of similar or greater complexity for further study.

For future work, we propose exploring how the attention mechanism of transformer‐based models can be leveraged to identify the most useful data features of multi‐sensor systems and inform their design choices. Further, we suggest evaluating the proposed pipeline to achieve unsupervised feature extraction with other types of multi‐sensor and spectral data in chemical sensing in order to confirm the expected general applicability of the concept.

## 4 Experimental Section

In this section the methodology used for experimental testing with the multi‐sensor system for chemical analysis is introduced. The implementation of this approach for analysis of sensor data response is described and details regarding data sets and corresponding learning tasks are provided.

### Image Representation of Multi‐Sensor Data

Encoding multi‐sensor array data into image representations is a crucial step to enable the use of vision models as feature extractors. There are different approaches that can be leveraged to transform numerical vectors into matrix representations, i.e., images, depending on the information content that needs to be preserved when switching from one modality to the other. For instance, the Gramian Angular Field (GAF) transformation has been applied in the context of time‐series data encoding into images.^[^ ^53^, ^54^ ^]^ GAF enables training of deep‐learning architectures or transfer learning from image‐encoding models. First, this approach requires transforming a normalized numerical vector $x=\left\{x_{1},x_{2},\text{…},x_{N}\right\}$ of length *N* in polar coordinates with angle and radius defined for each element *x*_*i*_ as:

(1) $$x_{i}^{p}=\left\{\begin{array}{c} \phi_{i}=\arccos\left(x_{i}\right)\\ r_{i}=\frac{i}{N} \end{array}\right.$$

Then, a Gram Matrix^[^ ^55^ ^]^ *G* is created starting from the angular components of all elements *x*_*i*_ with *i* = 0, … *N* and can be used directly for image representation.

(2) $$G=\left(\begin{array}{cccc} cos(\phi_{1}+\phi_{1}) & cos(\phi_{1}+\phi_{2}) & \cdots & cos(\phi_{1}+\phi_{N})\\ cos(\phi_{2}+\phi_{1}) & cos(\phi_{2}+\phi_{2}) & \cdots & cos(\phi_{2}+\phi_{N})\\ \vdots & \vdots & \ddots & \vdots \\ cos(\phi_{N}+\phi_{1}) & cos(\phi_{B}+\phi_{2}) & \cdots & cos(\phi_{N}+\phi_{N}) \end{array}\right)$$

The motivation behind the use of such approach is that the Gram Matrix operation preserves the inherent temporal dependency of a numerical vector. Thus, GAF was leveraged to transform the multi‐sensor responses into images. In particular, the 15 time‐dependent voltage signals were concatenated to form a unique vector with a spectrum‐like shape. For a 60 s liquid test, a vector of 900 (15 signals × 60 s recorded at 1 Hz) data points was generated. The GAF transformation was then applied to numerical vectors from all liquid measurements to obtain image representations. A schematic of such pre‐processing step is shown in Figure S1 (Supporting Information). This approach is proposed as an example that can provide a generic method to encode sensor array data into image representations. To enrich the images by emphasizing the intensity of the original sensor signals, an approach is proposed that requires multiplying the Gram Matrix by the original sensor response column‐wise and such transformation is denoted as GAF*. Results were obtained using the GAF implementation provided in the *pyts* Python package.^[^ ^56^ ^]^

### Pretrained Vision Models

A total of 12 pretrained vision models from the Hugging Face Hub^[^ ^57^ ^]^ were tested as feature extractors in the transfer learning pipeline. Two CNN‐based architectures, trained on the ImageNet‐1k dataset (1.3 million images, 1'000 classes), were selected: MobileNetV2^[^ ^48^ ^]^ and ResNet.^[^ ^47^ ^]^ Three different versions of the former architecture with varying depth multiplier (0.35, 1 and 1.4) and resolution (96 × 96 and 224 × 224) parameters were used and named *MobileNetV2 35 96*, *MobileNetV2 100 224* and *MobileNetV2 140 224* throughout this article. Three different variations of the ResNet architecture (*ResNet 18, 50*, and *101*) with varying number of layers and resolution 224 × 224 are also studied. Two types of transformer architectures were evaluated with this approach, the Vision Transformer (ViT)^[^ ^39^ ^]^ and BERT pretraining of Image Transformers (BeiT).^[^ ^58^ ^]^ Both models were pretrained on ImageNet‐21k (14 million images, 21'843 classes) at resolution 224 × 224 and fine‐tuned on ImageNet‐1k. For ViT, base and large variations were used as well as 224 × 224 and 384 × 384 resolutions with three configurations: *ViT base 224*, *ViT large 224* and *ViT base 384*. Regarding Beit, the same parameter configurations are evaluated: *BeiT base 224*, *BeiT large 224* and *BeiT base 384*.

### Sensor Data Featurization and Model Head Training

To train classification model heads and reconfigure the multi‐sensor system to address specific tasks, transfer learning is leveraged through features extracted with the twelve pretrained vision models. In particular, the pooled outputs obtained after applying the 12 architectures with frozen weights are extracted. Principal Component Analysis (PCA) was used to reduce the dimensionality of the feature space before training different classification model heads: Random Forest (RF), Linear Discriminant Analysis (LDA), K‐Nearest Neighbors (KNN), Support Vector Machines (SVM), Extra Trees (ET) and XGBoost (XGB). All model implementations considered are from the *sklearn* Python package^[^ ^59^ ^]^ with default hyperparameters for LDA, SVM, ET, and XGB. The number of trees for RF is fixed to 50,^[^ ^50^ ^]^ while a neighbor number equal to three was chosen for KNN. The number of principal components is specified within each section of the main text and varied depending on the types of task and validation. Generally, 20 principal components are used to train classification models for the identification tasks, comparison and benchmarking analysis. Results obtained for varying number of principal components are reported in the Supporting Information. To take into account the reduced size of training data in the few‐shot study, the number of principal components was reduced to 10. The performance comparison was carried out against handcrafted feature extraction reported previously.^[^ ^46^, ^50^ ^]^ Handcrafted features are subjected to the same pipeline as for features extracted from pretrained vision models, including PCA dimension reduction.

### Hierarchical Clustering

To validate the use of this processing pipeline a series of multi‐sensor measurements were run over various products (eight different samples of brewed coffee, nine fruit juices, eight mineral waters and seven bottled red wines) in a randomized order with an automated robotic setup. Tests were replicated for each product to produce a data set of 150 instances overall. Pretrained vision models were utilized for feature extraction and PCA was performed to reduce the dimensionality of our feature space. Hierarchical clustering was then investigated on the principal component scores to test the discriminatory ability of the multi‐sensor system combined with featurization from vision models. In particular, the agglomerative clustering approach^[^ ^60^ ^]^ implemented in the *scikit‐learn* ^[^ ^59^ ^]^ Python package was leveraged, using a predetermined number of groups equal to four, i.e. the number of major liquid classes in the data set (coffee, juice, water and wine). *Euclidean* and *ward* as distance and linkage metrics were chosen, respectively. The objective function of the *ward* linkage is based on a variance‐minimizing approach and provides more evenly sized clusters compared to other methods. Clustering was executed on 5, 10, 15, 20, and 25 principal components obtained after extracting features using the 12 pretrained models. The similarity between true and cluster‐assigned labels was evaluated by means of the Adjusted Rand Index (ARI), whose score approaches 0 for random labeling and equals 1 when clustered groups correspond to the true groups.

### Data Sets and Tasks

The methodology was applied to diverse downstream tasks to test the combination of portable sensors with pretrained models from other domains as a means to configure machine learning assisted systems for target use cases. Validation of the pipeline is conducted on small data sets, aiming at replicating practical scenarios in which a user would train such systems for personalized needs by means of manual measurements. All measurements were performed by fixing the dwell time in reference solution to 100 s and the testing time to 60 s. Details on sample preparation, data sets and validation methods are reported below.

### Wine Identification

The aim of this task is to train the system to recognize seven specific bottled Italian red wines. Four wines were produced in the Valpolicella area of the Veneto region and consist of mixtures of grape varieties such as Corvina, Rondinella, and Sangiovese. The other three wines were produced in Piedmont and were monovarietal samples of Barolo, Barbera and Nebbiolo. One of the Valpolicella samples was also used as reference and rinsing liquid. **Dataset**. Wine bottles were opened and samples thereof mounted on the robotic setup for direct testing. All samples were tested in randomized order and ten measurement replicates are performed for each wine, resulting in a total of 70 measurements. **Validation**. Models were validated by 50 repetitions of a random five‐fold split into training (80%) and test (20%) sets. Average and standard deviation of classification accuracy across the 50 replicates is reported in Figure 4 of the main text. Detailed results on varying number of principal components are reported in Figure S2 (Supporting Information).

### Protein Identification

The multi‐sensor system was tested for the recognition of six protein products based on pea (three samples) and soy (three samples) proteins produced by different vendors. Protein powders were dissolved in commercial mineral water (Acqua Panna) at 3.5% w/w concentration. Acqua Panna was used as reference and rinsing liquid for the automated testing. **Dataset**. Samples were tested repeatedly in randomized order producing 10 replicates for each product. The resulting data set comprised 60 measurements. **Validation**. The same validation procedure was used as for the wine identification task. Detailed results on varying number of principal components are reported in Figure S3 (Supporting Information).

### Sugar Type and Level Classification

The goal of this task is to teach the system to discriminate the type of sugar in a sample (*sucrose* or *glucose*) and approximate its concentration level (*low* or *high*). Sugar powders were dissolved in commercial mineral water (Acqua Panna) for testing at concentration levels of 2.5, 5, and 10% w/w. Pure Acqua Panna was used as reference and rinsing liquid for the automated testing. **Dataset**. Samples were tested in randomized order producing 10 replicates for each product, resulting in a total of 60 measurements. **Validation**. To test the ability to assess sugar type and level simultaneously, a leave‐one‐class‐out approach is followed on the 2.5 and 10% w/w extreme concentration levels for both sugar types. At each iteration, all the measurement replicates for the test class were removed from the training set and the 5% w/w was labeled as *low* (if the test sample has 2.5% w/w concentration) or *high* (if the test sample had 10% w/w concentration). Thus, models were tested on 40 unique measurements for the prediction of the correct class among *glucose low*, *glucose high*, *sucrose low* or *sucrose high*.

### Milk Source Classification

Our pipeline was evaluated after training to recognize the protein source of commercial milk products derived from *almond*, *cow*, *oat*, *pea*, or *soy*. Samples were obtained from different vendors or from the same vendor and exhibit varying composition such as sugar and fat content. **Dataset**. Twelve different products were tested: two samples each for almond, cow and soy, and three samples each for oat and soy. Each product was tested five times, resulting in a set of 60 measurements overall. Samples were tested in a randomized order and commercial mineral water (Acqua Panna) was used as reference and rinsing liquid. **Validation**. Models were tested using a leave‐one‐class‐out approach by removing all instances of the test product from the training data set and predicting the protein source among the five training classes. Thus, for each product tested there was at least one different product of the same protein source in the training set.

### Image Data Augmentation

The lack of training data is a key limiting factor that prevents extensive use of machine learning to process chemical sensor responses. Since this data transformation relies on image representations, techniques for image data augmentation can be leveraged to synthetically generate additional training data. Among the various approaches that have been studied in the literature, a *pixel‐wise* mixed augmentation method based on the *mixup* learning principle is proposed.^[^ ^61^, ^62^ ^]^ To augment the number of instances of each class *C*_*i*_ in an *N*‐class dataset (*C*_1_, *C*_2_, …, *C*_*N*_), a pair‐wise weighted mixing was performed of an image drawn from *C*_*i*_ and an image instance belonging to another class *C*_*j* ≠ *i*_. Thus, synthetic image samples were created from linear combinations of pixels associated with images of different classes. For any pixel $p_{C_{i}}^{xy}$ of a 2D image instance of class *C*_*i*_ and with coordinates (*x*, *y*), a synthetic pixel $s_{C_{i}}^{xy}$ is computed as:

(3) $$s_{C_{i}}^{xy}=a\cdot p_{C_{i}}^{xy}+b\cdot p_{C_{j\neq i}}^{xy}$$

with *a* + *b* = 1 and *a* > 0.5. The ratio *a*/*b* is referred to as the *mixing ratio* of the synthetic sample. Equation (3) was repeated for each pixel to generate a synthetic sample image. The same procedure was repeated within the training set multiple times with all the instances of the same class *C*_*i*_ and varying the type and instance of the second class *C*_*j* ≠ *i*_ for all *N* classes to generate a complete synthetic data set. In this case, such augmentation approach is preferred over geometric transformation (e.g., rotation or mirroring) due to the significance of the geometrical properties of the images generated with GAF/GAF* encoding. An example of the image mixing strategy is shown in Figure S4 (Supporting Information).

### Training Classification Heads with Few Instances

Portable chemical sensors enable testing samples outside of controlled environments (e.g. wet laboratories) and possibly from the sample source directly. However, in such remote chemical sensing scenarios, the capacity to collect comprehensive data sets across different sample categories may be limited. Therefore, portable sensing systems would require a user or machine to rely on few measurement instances to configure multi‐sensor systems for target applications. The effect of the number of training instances on multi‐sensor performance was explored by splitting a data set in training and testing sets, with the former comprising *k* shots per class and the latter comprising all the other measurements. Configurations of the pretrained vision models and classification model heads were tested in three, five and seven‐shot experiments. A random train/test split was repeated 50 times and the average accuracy and standard deviation obtained on the test set is reported. Similarly, a few‐shot study was also performed with image augmentation as described in the previous section. The training set was augmented such that each class resulted in 50 training instances including real and synthetic samples. The synthetic samples were generated with random choices of instances from the *k* training shots. Different values of the mixing ratio parameter (*a*/*b* in Equation 3) were tested: 0.95/0.05, 0.90/0.10, 0.85/0.15, 0.80/0.20, and 0.75/0.25. As in the case of a data set without data augmentation, a random train/test split was repeated 50 times and the average accuracy and standard deviation obtained on the test set is reported. For all the experiments, the first ten principal components of the image embeddings were used for model head training. **Statistical Analysis**. To compare model performances and estimate the impact of data augmentation on classification accuracy, a *t*‐test (*stats* module of the *scipy* Python package) is conducted on the distributions of the accuracy obtained over 50 model iterations with and without synthetic training samples. The *p*‐value of the statistical tests was reported to assess whether the mean of the two distributions were significantly different.

## Conflict of Interest

The authors declare no conflict of interest.

## Supporting information

Supporting Information

## Data Availability

The data set on the identification of seven bottled Italian red wines is available within the public code repository: https://github.com/IBM/AI‐assisted‐chemical‐sensing/tree/main/src/chemsense/vision/resources/datasets/red_wines
