# Supplementary material for: Digital Fingerprinting of Complex Liquids Using a Reconfigurable Multi‐Sensor System with Foundation Models
Source: Adv Sci (Weinh). 2024 Oct 7;11(44):2407513. doi: 10.1002/advs.202407513 (PMC11600221; doi:10.1002/advs.202407513)
Supplement: Supplementary file 1 — Supporting Information [file ADVS-11-2407513-s001.pdf]

ADVANCED SCIENCE
Open Access

Supporting Information

for *Adv. Sci.*, DOI 10.1002/advs.202407513

Digital Fingerprinting of Complex Liquids Using a Reconfigurable Multi-Sensor System with Foundation Models

*Gianmarco Gabrieli, Matteo Manica, Joris Cadow-Gossweiler and Patrick W. Ruch**

Supporting Information

Digital Fingerprinting of Complex Liquids Using a Reconfigurable Multi-Sensor System with Foundation Models

*Gianmarco Gabrieli, Matteo Manica, Joris Cadow-Gossweiler, Patrick W. Ruch**

Figure S1. Scheme of multi-sensor signal processing to generate image representation.

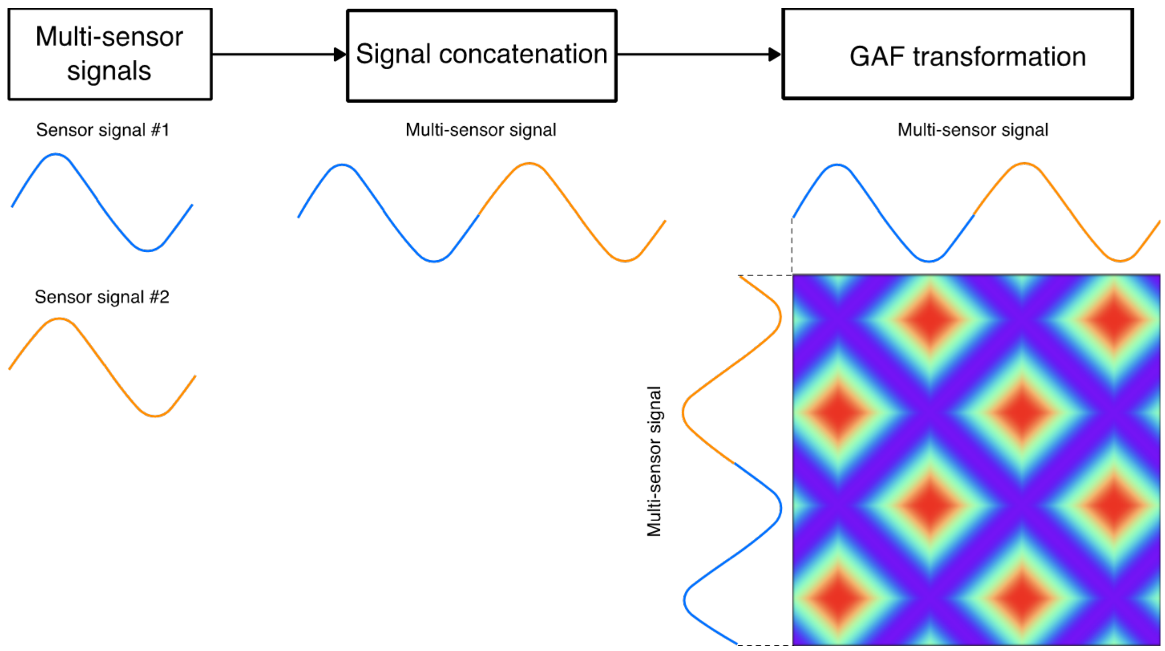

Figure S2: Five-fold cross-validation classification accuracy for **wine product identification (seven classes)** using 12 distinct pretrained vision models as feature extractors. In each case, the features are reduced to principal components and used to train six different classification model heads: Random Forest (RF), Linear Discriminant Analysis (LDA), K-Nearest Neighbors (KNN), Support Vector Machine (SVM), Extra Trees (ET) and XGBoost (XGB).

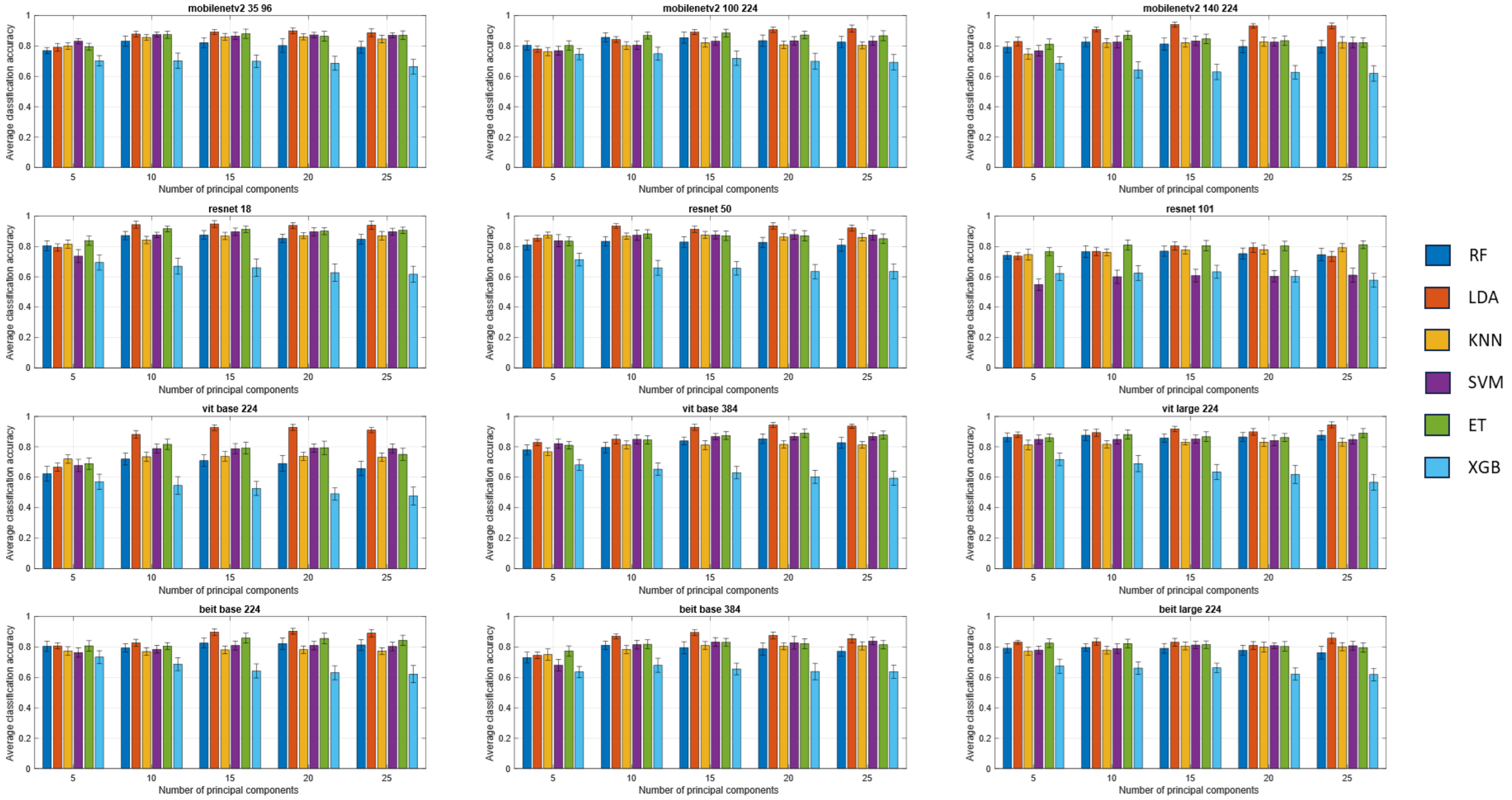

Figure S3: Five-fold cross-validation classification accuracy for **protein product identification (six classes)** using 12 distinct pretrained vision models as feature extractors. In each case, the features are reduced to principal components and used to train six different classification model heads: Random Forest (RF), Linear Discriminant Analysis (LDA), K-Nearest Neighbors (KNN), Support Vector Machine (SVM), Extra Trees (ET) and XGBoost (XGB).

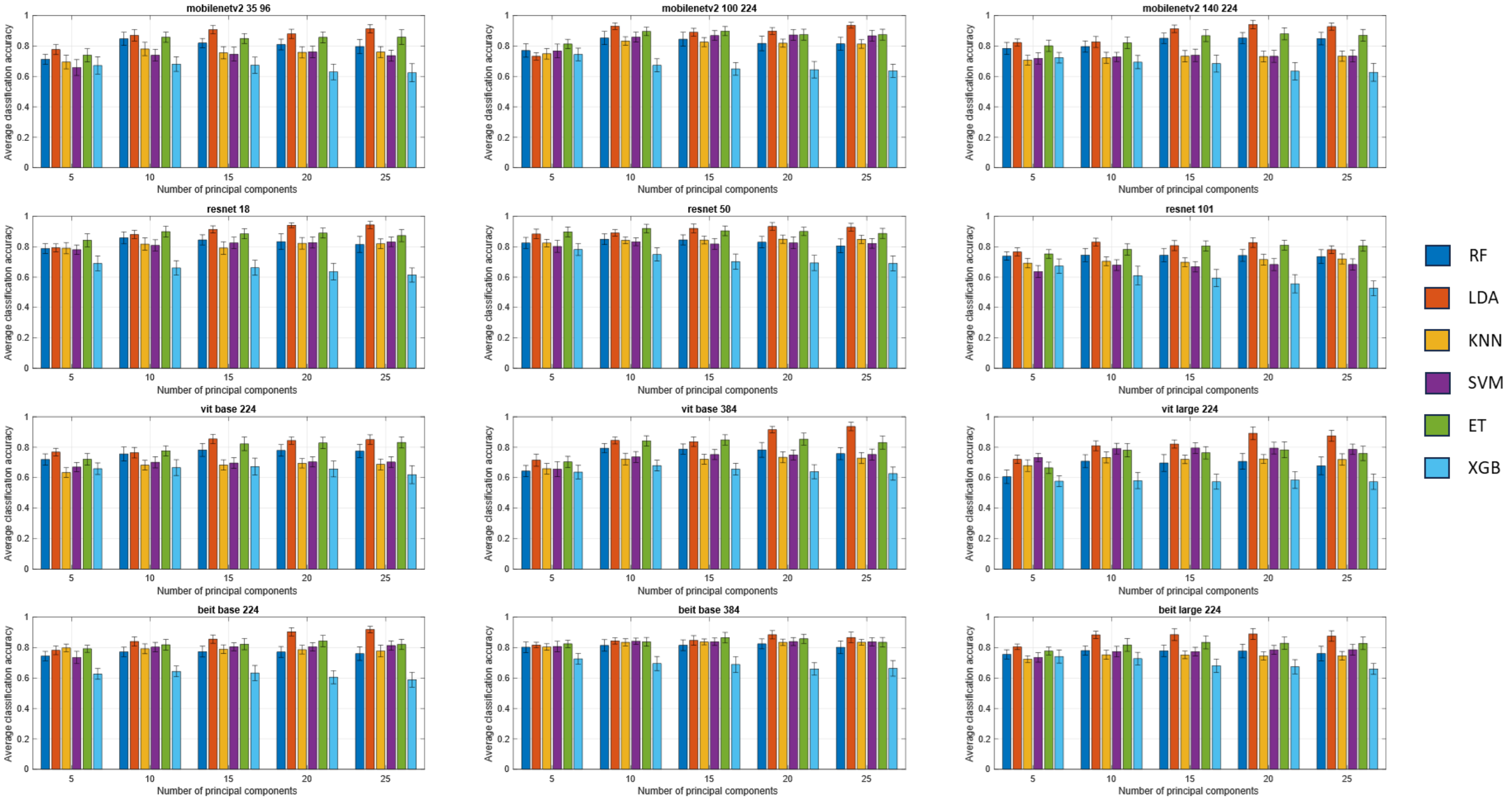

Figure S4: Example of pixel-wise image mixing for data augmentation pipeline. For a sample belonging to class *i*, synthetic samples can be generated by pair-wise weighted mixing with samples drawn from any class $j \neq i$. *a* and *b* are the weights used for image mixing, with $a > 0.5$ and $a + b = 1$.

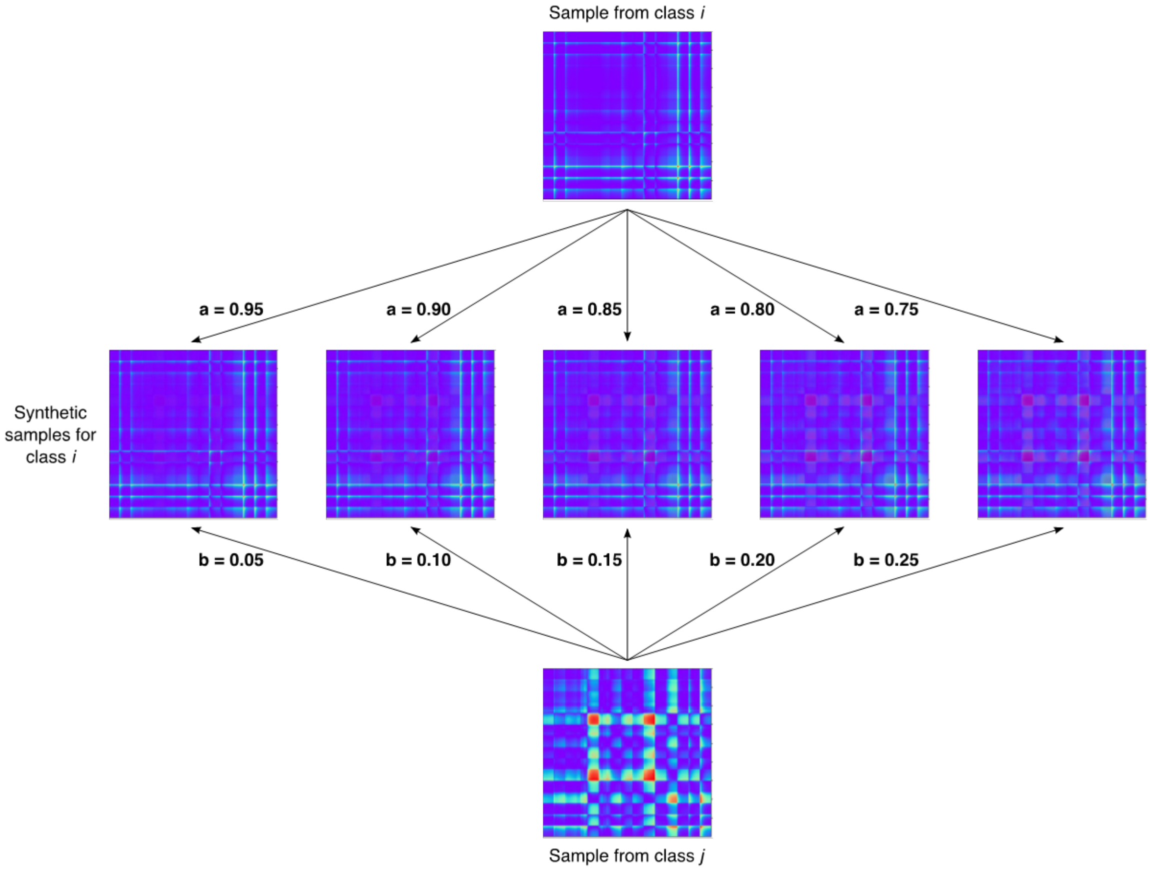

Figure S5: Average classification accuracy for identification of bottled Italian red wines in a three-shot training scenario after image data augmentation (cf. Experimental Section in the main text) by varying the mixing ratio parameter. Results are reported using twelve pretrained vision models as feature extractors and six classification model heads (RF, LDA, KNN, SVM, ET and XGB) after dimension reduction to 10 principal components. The best performances were obtained with features extracted using MobileNetV2 35 96, ResNet 18, ResNet 50 and ViT large 224 pretrained vision models regardless of the classification model head. The image mixing ratio parameter of the data augmentation method (cf. Experimental section 4) did not affect considerably performances for the model heads that demonstrated superior performances (LDA, KNN, SVM and ET). Instead, using the strongest image mixing (0.75/0.25), the performance of the XGB model head were improved up to 13%. However, XGB and RF resulted to be the least performing model heads with accuracy scores consistently lower than for the other four model heads.

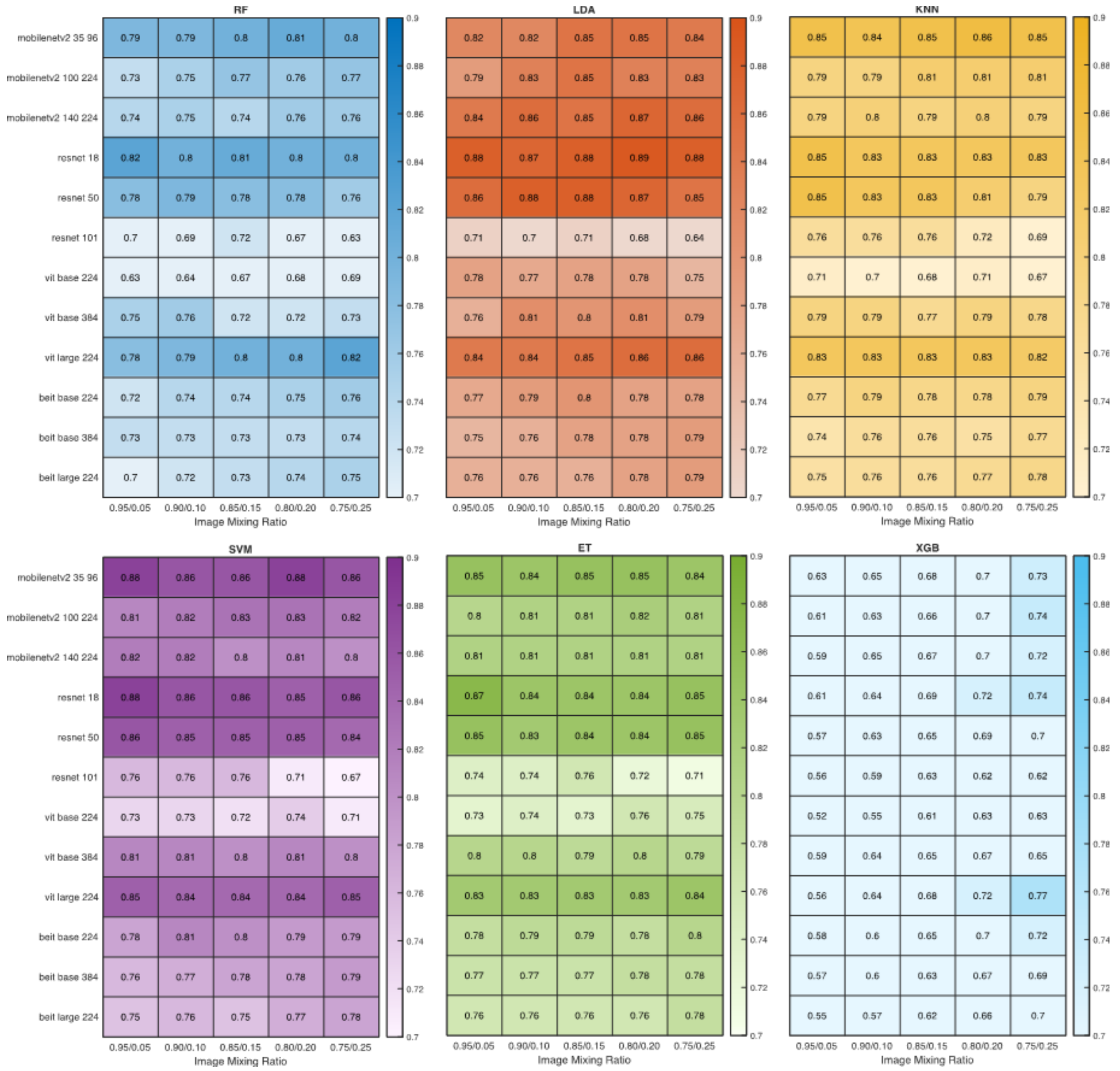

Table S1: Comparison of average classification accuracy for protein product identification using handcrafted features against the accuracy achieved using fingerprints obtained from pretrained vision models as feature extractors (five-fold validation and 20 principal components). Results are presented for two image encoding approaches (GAF and GAF*, cf. Experimental section 4). MN = MobileNetV2, RN = ResNet, VB = ViT base, VL = ViT large, BB = BeiT base, BL = BeiT large. Best results within the standard deviation uncertainty are highlighted in bold. Models trained with pretrained vision model features consistently outperform handcrafted features for two (KNN and SVM) out of six classification heads. The best performance is obtained combining GAF* encoding, ViT base 384 features and LDA model head. We achieve a classification accuracy of 95.5 ± 2.4% that is comparable to the best metric obtained with expert-curated features.

Features		RF	LDA	KNN	SVM	ET	XGB
Expert-curated		**96.0±1.8**	**98.8±1.3**	70.4±3.8	57.8±4.6	**97.4±1.2**	**82.8±4.1**
GAF	MN 35 96	81.0±3.5	88.0±3.0	75.8±3.7	76.1±3.7	85.7±3.6	62.9±5.1
	MN 100 224	81.7±4.9	89.8±2.3	81.9±2.7	**87.3±3.5**	87.5±3.6	64.4±5.4
	MN 140 224	85.2±3.6	94.2±2.8	73.1±3.6	73.2±4.0	88.0±4.0	63.5±5.7
	RN18	83.4±5.1	94.2±1.6	**82.3±3.8**	**82.8±3.6**	89.0±3.3	63.6±5.4
	RN50	83.0±3.8	93.4±2.6	**85.0±2.8**	**82.6±3.9**	90.1±2.8	69.3±5.0
	RN101	74.2±3.9	82.8±3.2	71.6±3.6	68.3±4.0	81.0±3.2	55.6±5.0
	VB224	77.9±3.8	84.4±2.4	69.4±3.1	70.3±3.3	82.8±3.7	65.7±5.2
	VB384	78.3±4.9	91.6±2.0	73.4±3.6	75.0±3.3	85.3±4.0	63.9±4.6
	VL224	70.7±5.2	89.2±4.2	72.3±2.9	79.4±3.9	78.3±5.2	58.4±5.5
	BB224	77.2±3.6	90.4±2.7	78.6±2.8	80.5±2.8	84.3±3.7	60.6±4.3
	BB384	82.5±3.3	88.4±2.7	**83.5±2.1**	**84.0±2.7**	85.8±3.0	66.0±4.1
	BL224	77.8±4.5	88.9±3.6	74.6±2.9	78.5±2.9	82.9±4.1	67.4±4.7
GAF*	MN 35 96	85.2±4.2	93.2±1.9	**88.0±2.8**	**83.6±3.3**	89.1±3.1	70.0±4.2
	MN 100 224	88.1±4.1	95.2±1.9	73.0±3.2	77.4±3.7	90.6±3.2	68.6±4.0
	MN 140 224	86.9±4.7	95.4±2.0	75.8±3.7	71.1±4.2	86.6±4.0	71.1±4.5
	RN18	84.2±3.8	94.6±2.6	74.3±3.0	66.0±5.2	87.5±4.2	70.2±4.8
	RN50	84.1±4.3	87.8±2.4	78.9±3.1	**83.4±2.6**	85.4±3.6	66.8±4.7
	RN101	76.8±4.3	82.1±4.1	74.7±4.2	79.5±3.5	83.1±3.1	67.3±4.2
	VB224	81.3±4.4	94.0±2.5	76.7±2.6	76.1±4.1	83.8±3.8	62.2±5.5
	VB384	80.1±3.3	**95.5±2.4**	**82.0±3.4**	79.1±3.4	86.1±3.6	64.8±5.0
	VL224	78.5±4.6	92.4±2.5	72.4±3.0	73.3±3.8	81.3±3.6	64.4±5.3
	BB224	80.5±4.6	91.8±3.1	71.3±2.7	73.1±3.2	82.9±3.3	61.4±4.0
	BB384	85.0±2.9	95.0±1.9	79.8±2.3	73.0±3.8	88.7±3.3	60.5±6.3
	BL224	82.0±3.8	91.7±3.0	78.7±2.9	72.3±4.6	85.1±2.7	72.9±5.3

Table S2: Comparison of classification accuracy obtained on the test set for sugar and milk categorization tasks using handcrafted features against the accuracy achieved using fingerprints obtained from pretrained vision models as feature extractors (five-fold validation and 20 principal components). Results are presented for two image encoding approaches (GAF and GAF*, cf. Experimental section 4). MN = MobileNetV2, RN = ResNet, VB = ViT base, VL = ViT large, BB =BeiT base, BL = BeiT large. For sugar sample categorization based on type and concentration level, perfect classification is obtained with handcrafted features and by combining GAF* encoding, ViT large 224 features and LDA model head. For milk protein source identification, the best accuracy obtained with expert-curated feature extraction is 80.0% while the best accuracy for pretrained feature extractors is 95.0% obtained by combining GAF* encoding, MobileNetV2 140 224 features and LDA model head.

		Sugar Level and Type						Milk source					
Features		RF	LDA	KNN	SVM	ET	XGB	RF	LDA	KNN	SVM	ET	XGB
Expert-curated		**92.5**	**100**	**92.5**	**100**	85.0	70.0	61.7	80.0	**80.0**	78.3	66.7	48.3
GAF	MN 35 96	57.5	65.0	90.0	75.0	70.0	62.5	65.0	81.7	65.0	73.3	73.3	51.7
	MN 100 224	70.0	90.0	87.5	62.5	80.0	52.5	61.7	81.7	70.0	**81.7**	71.7	63.3
	MN 140 224	45.0	77.5	82.5	80.0	60.0	47.5	76.7	71.7	76.7	68.3	68.3	53.3
	RN18	67.5	95.0	87.5	67.5	82.5	57.5	71.7	80.0	73.3	70.0	78.3	63.3
	RN50	70.0	80.0	82.5	87.5	80.0	67.5	65.0	78.3	**80.0**	78.3	73.3	65.0
	RN101	47.5	72.5	57.5	65.0	52.5	40.0	60.0	83.3	68.3	61.7	71.7	61.7
	VB224	50.0	77.5	67.5	75.0	57.5	52.5	63.3	78.3	71.7	65.0	**81.7**	43.3
	VB384	80.0	75.0	87.5	87.5	77.5	72.5	68.3	76.7	75.0	75.0	76.7	60.0
	VL224	77.5	55.0	72.5	82.5	72.5	55.0	63.3	73.3	73.3	73.3	63.3	**66.7**
	BB224	45.0	67.5	52.5	55.0	60.0	42.5	51.7	71.7	66.7	76.7	71.7	50.0
	BB384	77.5	80.0	67.5	82.5	**92.5**	60.0	48.3	73.3	51.7	55.0	48.3	53.3
	BL224	57.5	97.5	70.0	67.5	72.5	37.5	50.0	76.7	63.3	68.3	60.0	58.3
GAF*	MN 35 96	57.5	95.0	57.5	70.0	72.5	57.5	66.7	80.0	68.3	68.3	70.0	53.3
	MN 100 224	52.5	87.5	87.5	90.0	82.5	30.0	73.3	83.3	70.0	71.7	70.0	60.0
	MN 140 224	57.5	85.0	87.5	92.5	80.0	67.5	70.0	**95.0**	**80.0**	76.7	68.3	50.0
	RN18	70.0	85.0	80.0	85.0	75.0	67.5	61.7	76.7	71.7	75.0	68.3	51.7
	RN50	57.5	87.5	80.0	85.0	67.5	52.5	**80.0**	68.3	65.0	36.7	80.0	46.7
	RN101	57.5	77.5	50.0	52.5	65.0	62.5	71.7	75.0	56.7	38.3	66.7	61.7
	VB224	60.0	62.5	62.5	85.0	75.0	57.5	70.0	68.3	66.7	63.3	71.7	53.3
	VB384	67.5	85.0	75.0	72.5	67.5	47.5	46.7	71.7	68.3	68.3	58.3	48.3
	VL224	80.0	**100**	72.5	85.0	**90.0**	45.0	61.7	76.7	65.0	70.0	78.3	48.3
	BB224	57.5	87.5	77.5	82.5	82.5	65.0	60.0	90.0	65.0	76.7	70.0	33.3
	BB384	67.5	82.5	82.5	80.0	70.0	55.0	56.7	73.3	61.7	73.3	70.0	45.0
	BL224	65.0	60.0	72.5	75.0	67.5	60.0	45..0	75.0	71.7	53.3	63.3	50.0
